# Anti–PD-1/PD-L1 Blockade Immunotherapy Employed in Treating Hepatitis B Virus Infection–Related Advanced Hepatocellular Carcinoma: A Literature Review

**DOI:** 10.3389/fimmu.2020.01037

**Published:** 2020-05-28

**Authors:** Bin Li, Cong Yan, Jiamin Zhu, Xiaobing Chen, Qihan Fu, Hangyu Zhang, Zhou Tong, Lulu Liu, Yi Zheng, Peng Zhao, Weiqin Jiang, Weijia Fang

**Affiliations:** ^1^Department of Medical Oncology, The First Affiliated Hospital, School of Medicine, Zhejiang University, Hangzhou, China; ^2^Department of Oncology, Henan Cancer Hospital, The Affiliated Cancer Hospital of Zhengzhou University, Zhengzhou, China

**Keywords:** immunotherapy, hepatocellular carcinoma, hepatitis B virus, programmed cell death protein 1, programmed death-ligand 1, tumor microenvironment

## Abstract

Hepatitis B virus (HBV) infection is regarded as the main etiological risk factor in the process of hepatocellular carcinoma (HCC), as it promotes an immunosuppressive microenvironment that is partially mediated by the programmed cell death protein 1 (PD-1)/programmed death-ligand 1 (PD-L1) signaling pathway. The tumor microenvironment (TME) of HBV–related HCC is indeed more immunosuppressive than microenvironments not associated with viruses. And compared to TME in hepatitis C virus (HCV) infected HCC, TME of HBV-related HCC is less vascularized and presents different immune components resulting in similar immunosuppression. However, few studies are focusing on the specific side effects and efficacy of PD-1/PD-L1 blockade immunotherapy in HBV-related HCC patients, as well as on the underlying mechanism. Herein, we reviewed the basic research focusing on potential TME alteration caused by HBV infection, especially in HCC patients. Moreover, we reviewed PD-1/PD-L1 blockade immunotherapy clinical trials to clarify the safety and efficacy of this newly developed treatment in the particular circumstances of HBV infection. We found that patients with HBV-related HCC displayed an acceptable safety profile similar to those of non-infected HCC patients. However, we could not determine the antiviral activity of PD-1/PD-L1 blockade because standard anti-viral therapies were conducted in all of the current clinical trials, which made it difficult to distinguish the potential influence of PD-1/PD-L1 blockade on HBV infection. Generally, the objective response rates (ORRs) of PD-1/PD-L1 blockade immunotherapy did not differ significantly between virus-positive and virus-negative patients, except that disease control rates (DCRs) were obviously lower in HBV-infected HCC patients.

## Background

Liver cancer was predicted to be the sixth most commonly diagnosed cancer and the fourth-leading cause of cancer death in 2018, with about 841,000 new cases and 782,000 deaths annually (1). Among the various types of primary malignant hepatic tumors, hepatocellular carcinoma (HCC) is the most common, accounting for roughly 75–85% of cases (1, 2).

Chronic hepatitis B virus (HBV) infection is always regarded as a primary risk factor for the development of HCC and accounts for at least 50% of HCC cases worldwide (3, 4). The world is of a high prevalence of HBsAg, especially eastern Asian. At least 120 million Chinese people carry HBsAg which makes China a highly endemic area, and the second-greatest proportion of cancer deaths is attributable to HBV (5–7). Notably, the potential risk of developing HCC is dozens of times higher for chronic HBV carriers compared with the uninfected population (8).

Programmed cell death protein 1 (PD-1), an immunosuppressive molecule expressed in B cells, T cells, dendritic cells and natural killer (NK) T cells to suppress anticancer immunity, has been shown to be correlated with the course of HCC and with HBV infection (9–12). Nowadays, anti-PD-1/programmed death-ligand 1 (PD-L1) pathway blockade has become a promising and favorable immunotherapy for adjusting host immune responses and inhibiting the development of various types of tumors (13–17). However, HBV infection exerts complex biological effects on the tumor microenvironment (TME), which probably affects the efficacy of immunotherapy to a certain extent. Unless the safety and efficacy of anti-PD-1/PD-L1 therapy in HBV-infected HCC patients can be confirmed, the role that this immune-adjusted therapeutic strategy could play in HBV infection related HCC might not be clarified.

## HBV Infection related liver immune microenvironment alteration

### Liver Tolerogenic Mechanisms: Natural Status

In the human liver, normal biological processes involve a large amount of antigen exposure. The existence of antigens could lead to inactivation, tolerance, and apoptosis of T cells, protecting the liver from autoimmune damage of continuous immune stimulation (18). Previous studies have revealed several tolerogenic mechanisms, including a porous layer established to isolate hepatic tissue from the blood (19) and the release of the immunosuppressive cytokines interleukin 10 (IL-10) or transforming growth factor-beta (TGF-β) from liver dendritic cells, liver sinusoidal endothelial cells (LSECs), and Kupffer cells (20). Additionally, the liver could generate antigen-specific Cluster of Differentiation 4 (CD4^+^)/forkhead box P3 (FOXP3^+^) regulatory T cells (Tregs) (21) and downregulate the expression of B7-1/B7-2 on the surfaces of LSECs, which would limit the ability of antigen-presenting cells (APCs) to activate CD4^+^ T cells (22). The immune checkpoint pathways, B7-CD28/cytotoxic T lymphocyte–associated antigen-4 (CTLA-4) and PD-1/(PD-L1, PD-L2), also contribute to natural liver immune tolerance (19, 23). Furthermore, both harmless and harmful antigens could protect the liver from autoimmune damage via inherent immune tolerance or escape mechanisms (22).

### HBV Infection–Based TME

HCC is regarded as a highly heterogeneous disease of varying immune microenvironments between the tumor and adjacent tissues (24). Chronic inflammation is generally considered to be the ongoing expression of different cytokines and recruitment of immune cells to troubled regions (22). HBV virus infection induces immunosuppression, and then peripheral immune tolerance as the chronic infection progresses; finally, it mediates oncogenesis, due to impaired immune surveillance (25). In chronic viral hepatitis, immune inhibitory checkpoints, including PD-1/PD-L1, CTLA-4, and T-cell immunoglobulin and mucin domain-3 (TIM-3), play essential roles in immunosuppression by downregulating the responses of T cells (22).

**Figure 1:**
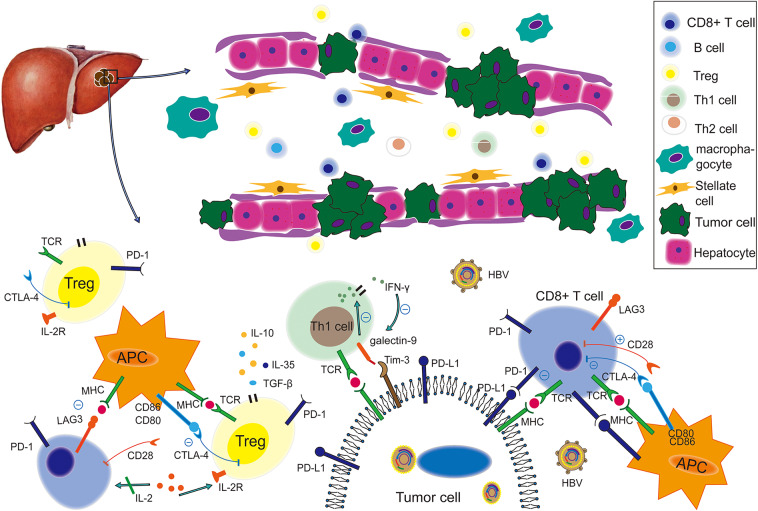
The immune landscape of the HBV-based tumor microenvironment. Under virus infection conditions, the immune status of an HCC-bearing host becomes more immunosuppressive and is characterized by weakening of co-stimulatory signal, enhancement of co-inhibitory signals, functional impairment, decreased quantity of tumor-killing T cells, such as CD8^+^ and enrichment of Tregs. Therein, the PD-1/PD-L1 pathway plays a suppressive role to produce cancer immune escape, while the LAG3/MHC-II, Tim-3/galectin 9, and CTLA-4/(CD80, CD86) pathways also contribute to this process. This figure was drawn with Adobe Illustrator CS5. TCR, T-cell receptor; MHC, major histocompatibility complex; APC, antigen-presenting cell; PD-1, programmed cell death protein 1; PD-L1, programmed death-ligand 1; CTLA-4, cytotoxic T lymphocyte associated antigen-4; Tim-3, T-cell immunoglobulin and mucin domain-3; *LAG3*, lymphocyte activation gene 3; TGF-β, transforming growth factor beta.

Figure 1 illustrates the immune landscape for HBV-related TME in HCC. CD8^+^ T cells provide a vital antitumor response in the surrounding HCC microenvironment. The gradually increasing frequencies of circulating CD8^+^ T cells expressing PD-1 were reported to be relevant to the progression of HBV-related cirrhosis to HCC. Apoptosis of CD8^+^ T cells was also promoted by hepatoma cells via PD-L1 upregulation (26). Of note, transcriptional analysis has discovered an extra cytotoxic phenotype of CD8^+^ tumor-infiltrating lymphocytes in HCC patients with undetectable serum levels of HBV (27). In addition, HBV replication was also found to be associated with a higher proportion of HBV-specific CD8^+^ T cells. TIM-3 inhibits Th_1_ responses and expresses effective cytokines such as tumor necrosis factor (TNF) and interferon-gamma (INF-γ) (27, 28). A recent study revealed an inverse association between TIM-3 expression levels and clinical outcomes in HBV-infected HCC (29): The proportion of TIM-3^+^ CD8^+^ T cells in tumor tissues from HBV^+^ patients was much higher than in those from HBV^−^ patients (CD8^+^: 15% vs. 2%), and the TIM-3/galectin-9 signaling pathway could mediate T-cell functional exhaustion in HBV-infected patients. Notably, higher expression of PD-1 and lymphocyte-activation gene 3 (*LAG3*), as well as lower expression of CD28 and CD127, were commonly found in tumor-infiltrating CD8^+^ T cells of HBV-related HCC patients (30–32).

In the TME, Tregs play an immunosuppressive role by producing cytokines, such as IL-10, IL-35, and TGF-β, and by inhibiting Th_1_ or Th_2_ cell activation (33). Frequencies of Tregs were higher in HBV^+^ HCC than HBV^−^ HCC patients. Decreased PD-1 expression and increased IL-10/TGF-β secretion of CD4^+^ CD25^+^ Tregs were found in HBV^+^ HCC patients (34). However, researchers have also clarified that the increased PD-1 expression in Tregs from HBV^+^ patients indicated a more suppressive and exhausted immune condition (35). In the studies mentioned above, Tregs had different levels of PD-1 expression but resulted in similarly suppressive immune conditions in HBV-based TMEs. A next-generation sequencing (NGS) analysis (35) revealed that Tregs isolated from HBV-infected HCC had distinct transcriptomic signatures containing 289 differentially expressed genes. For instance, upregulated expression of *FOXP3*, and other genes involved in the IL-10 pathway including interleukin 1 receptor type 1 (*IL1R1*) and TNF receptor superfamily member 1B (*TNFRSF1B*) were found in Tregs from HBV-infected HCC. And *LAG3*, essential for suppressive activity, was also enriched. *CTLA-4* is another immunosuppressive molecule, and an *in vitro* experiment suggested that CD4^+^ CD25^+^ Tregs isolated from peripheral-blood mononuclear cells could upregulate the expression of *CTLA-4* after being co-cultured with stably HBV-transfected human hepatoma cell lines (36).

Resident memory T cells (Trms) were also enriched in the TME of HBV-related HCC. In these cells, exhaustion markers, such as *PD-1, LAG3, TIM-3*, and *CTLA-4*, were more highly expressed, and expression of multiple pro-inflammatory markers, including IFN-γ, IL-17a, granzyme B, and TNF-α, was lower (35). Trms were more function-suppressive and exhausted in virus-positive HCC than those in virus-free HCC. In addition, the immunosuppressive status of insufficient TNF-α and IFN-γ expression did not change in PD-1–expressing Trms from HBV-related HCC patients after phorbol myristate acetate (PMA)/ionomycin stimulation (35).

The percentages of myeloid-derived suppressor cells (MDSCs) were found to be higher in HBV-infected HCC patients (37, 38). MDSCs promoted a continuous immune-suppressive effect, along with persistent HBV infection and HCC progression (39). These cells could regulate the immune response in chronic HBV–infected patients via PD-1 induced IL-10 (40). They could also induce CD4^+^ CD25^+^ FOXP3^+^ Tregs and exhausted CD8^+^ T cells and inhibit NK cells in HCC (41, 42).

In the past, young patients were regarded as immunotolerant for HBV infection. A study aiming to explore the T-cell tolerance immune profile of young HBV-infected persons found that effector/inflammatory cytokines produced by T cells, including IFN-γ, TNF- α, IL-17A, and IL-22, were significantly higher in such patients with chronic hepatitis B infection than in healthy individuals (43). The frequency of PD-1^+^ CD127 low-CD8^+^ T cells increased with age in patients infected with chronic hepatitis B, and a less-compromised HBV-specific T-cell repertoire was also increasingly detected in young patients compared with adult patients with chronic infection (43). Therefore, the long process of infection may lead to a progressive state of T-cell exhaustion and excessive production of immunosuppression-related cytokines.

Blocking the corresponding immunosuppressed signal could recover the function of immune cells and improve immune reaction. One study found that PD-L1 blockade yielded an ~2-fold increase in HBV-specific T cells, vs. incubation with isotype control (12, 44). Not only was HBV-specific CD4^+^ and CD8^+^ T-cell response improved upon incubation with anti–PD-L1, but IL-2 and IFN-γ secretion and proliferation of HBV-specific CD8^+^ cells were likewise recovered via PD-L1 blockade. In addition, HBcAg-specific IFN-γ production was increased after anti–PD-1 monoclonal antibody used in intrahepatic T lymphocytes (45). Immunosuppression caused by PD-1^+^ Tregs could be reversed with PD-1 blockade (35), and so could the exhaustion of intrahepatic CD8^+^ T cells by combined PD-1 and CTLA-4 inhibition (26).

Similar to HBV, HCV infection blocked or altered host immune responses, and resulted in chronic inflammation status (46). In HCV related HCC, tumor tissue was reported to be more vascularized than those of HCC with HBV infection (47, 48). A recent research revealed that different tumor-infiltrating leukocytes composition in different subtypes of HCC. M0 macrophage and neutrophil cells were found to predominate in HCV^+^ tumor, and resting memory CD4^+^ T cells, activated memory CD4^+^ T cells, activated NK cells, resting dendritic cells, and resting mast cells were significantly higher in HBV^+^ tumor tissue contrast with non-tumor samples (49). The significantly elevated MDSCs were found in HCV^+^ HCC especially in advanced stage HCC, and positively correlated with HCV viral load (50). Similarly, the proportion of Treg cells, acting the immunosuppressed role, was also significantly higher in HCV related HCC, especially in the recurrence subset (51). CD4^+^ T cells and T-cell stimulatory activity of dendritic cells were significantly decreased in HCV related HCC patients (50, 52). The increased CD4^+^CD57^+^ T cells in peripheral blood lymphocytes were associated with tumor progression and had a significant inverse correlation with IFN-γ-producing capability (53). Additionally, HCV-HCC patients generated significant higher values for both IL-10, IL-18, and TGF-β (54, 55). HCV-HCC patients present immunosuppressed status like HBV infected HCC, and future researches should be carried out to explore the interplay and specific function of immune cells in TME and have transcriptome analyses of represented cell subsets using advanced single-cell sequencing technology.

In summary, the TME of HBV-related HCC is considered to be more immunosuppressive and exhausted than that of non HBV-related HCC (35). As the hotspot of immunotherapy, the PD-1/PD-L1 pathway has gradually become the most important therapeutic target for rescuing immune cells and avoiding tumor progression.

## Efficacy Evaluation of Anti–PD-1/PD-L1 Therapy

### Efficacy of PD-1/PD-L1 Inhibitors in HBV-related HCC

Currently, clinical trials are ongoing to evaluate the efficacy of PD-1/PD-L1 inhibitors as monotherapy or part of combined therapeutic strategies in HCC patients (Table 1). In HCC patients treated with anti–PD-1/PD-L1 monotherapy, objective response rates (ORRs) ranged from 8 to 20%, and disease control rates (DCRs) ranged from 33 to 73%, except one was 10% (Table 1). With PD-1/PD-L1 based combination therapy, patients achieved higher ORRs and DCRs (ORR range, 20–50%; DCR range, 49–100%).

**Table 1:** PD-1/PD-L1 blockade efficacy in HBV^+^ HCC and total HCC patients.

				**Objective response**		**Disease control**		
**Drug**	**ClinicalTrials.gov number**	**Line of therapy**	**Target**	**HBV^**+**^ HCC**	**Total HCC**	**HBV^**+**^ HCC**	**Total HCC**	**References**
Nivolumab	NCT02576509	1	PD-1		57 (15%)		187 (50%)	(56)
Nivolumab	NCT01658878 (escalation phase)	1/2	PD-1	1 (7%)	7 (15%)		28 (58%)	(57)
Nivolumab	NCT01658878 (expansion phase)	1/2	PD-1	7 (14%)	42 (20%)	28 (55%)	138 (64%)	(57)
Nivolumab	—	—	PD-1	—	6 (8%)	—	30 (39%)	(58)
Nivolumab	—	2	PD-1	—	4 (12%)	—	12 (35%)	(59)
Pembrolizumab	NCT02702414	2	PD-1	—	18 (17%)	—	64 (62%)	(60)
Pembrolizumab	NCT02702401	2	PD-1	—	51 (18%)	—	—	(61)
Cemiplimab	NCT02383212	1	PD-1	—	5 (19%)	—	19 (73%)	(62)
BGB-A317	NCT02407990	—	PD-1	—	1 (10%)	—	7 (10%)	(63)
SHR-1210	NCT02989922	≥1	PD-1	—	32 (15%)	—	96 (44%)	(64)
Durvalumab	NCT01693562	—	PD-L1	0 (0%)	4 (10%)	1 (11%)	13 (33%)	(65)
Durvalumab + tremelimumab	NCT02519348	1/2	PD-L1 + CTLA-4	1 (9%)	10 (25%)	5 (45%)	23 (58%)	(66)
Durvalumab + tremelimumab	NCT02821754	≥ 1	PD-L1 + CTLA-4	—	2 (20%)	—	6 (60%)	(67)
Nivolumab + ipilimumab	NCT01658878	—	PD-1 + CTLA-4	—	46 (31%)	—	146 (49%)	(68)
Atezolizumab + bevacizumab	NCT02715531	1	PD-L1 + VEGF	11 (31%)	23 (32%)	—	56 (77%)	(69)
Atezolizumab + bevacizumab	NCT03434379	1	PD-L1 + VEGF	—	89 (27%)	—	240 (74%)	(70)
Pembrolizumab + lenvatinib	NCT03006926	—	PD-1 + VEGF	3 (50%)	11 (42%)	6 (100%)	26 (100%)	(71)
SHR-1210 + apatinib	NCT02942329	—	PD-1 + VEGF	8 (50%)	8 (50%)	15 (94%)	15 (94%)	(72)

*Data are n (%). HBV^+^ HCC, HBV-infected HCC; PD-1, programmed cell death protein 1; PD-L1, programmed death-ligand 1; CTLA-4, cytotoxic T lymphocyte associated antigen-4; VEGF, vascular endothelial growth factor*.

Irrespective of line of therapy, ORRs of advanced HCC with nivolumab were 15–20%, and a substantial reduction in tumors from baseline was also observed (CheckMate 040) (57). The trial suggested the ORRs of patients infected with HCV, HBV and without viral hepatitis were 20, 14, and 22%, respectively, but these acquired data were not powered for statistical comparisons. In the dose-expansion phase, 6- and 9-month overall survival rates were 84 and 70%, respectively, in HBV-infected patients (57). As for the total study population, 6- and 9-month overall survival rates were, respectively, 83 and 74%. Besides, a part of researches supported the off-label use of nivolumab after failure or intolerance of sorafenib treatment or disease progression (73). A subsequent study of nivolumab as first-line therapy (Checkmate 459) not only confirmed the findings observed with second-line nivolumab, but also demonstrated improved quality of life and reduced treatment burden in advanced HCC. As a primary endpoint, consistent effect on overall survival was also observed in advanced HCC with nivolumab and seemingly profitted more in predefined subgroup of HBV infection (56).

Pembrolizumab (Keytruda; prescribing information at https://www.accessdata.fda.gov/drugsatfda_docs/label/2017/125514s015lbl.pdf), another anti-PD-1 monoclonal antibody, was used in a phase 2 trial (KEYNOTE-224) involving advanced-HCC patients who had previously been treated with sorafenib. ORR, median progression-free survival rate, and 12-month overall survival rate were 17%, 12.9 months and 54%, respectively (60). Of note, in the subgroup analysis of viral infection, comparable reductions of target lesions from baseline were observed in patients with or without HBV or HCV infection at the rates of 57 and 58%, respectively (60). In phase 3 study (Keynote 240), ORR was 18% and 4% for pembrolizumab vs. placebo at final analysis (one-sided *P* < 0.001), and in Asian subgroup, ORR was 21% for pembrolizumab and 2% for placebo (*P* < 0.001). Notably, HBV infection status was much higher in Asian subgroup (Asian: 51% vs. overall: 25%) (61, 74).

In China, 217 HCC patients intolerant of first-line treatment or with progressive disease were treated with SHR-1210 (camrelizumab, a fully humanized anti-PD-1 immunoglobulin G4 [IgG4] monoclonal antibody) as second-line treatment (64). Of all of the subjects, 32 (15%) achieved partial response, and 18 patients remained effective at the cutoff point. The 6-month overall survival rate was 74%; the median overall survival was not reached by the time of preliminary analysis. It is noteworthy that 89% of patients were infected with HBV and most had serious disease status, including extrahepatic metastasis and alpha-fetoprotein (AFP) ≥ 400 ng/mL. SHR-1210 monotherapy showed clinical efficacy similar to that of nivolumab and pembrolizumab in this trial. In another open-label, dose-escalation/expansion study from China, 94% of HCC patients achieved a state of disease control and 51% of patients achieved 6-month progress-free survival using SHR-1210 combined with apatinib (72). Interestingly, these enrolled HCC patients were all HBV-infected and with heavy tumor burdens, suggesting that the combination therapy likely had greater efficacy than single-agent immunotherapy (Table 1).

Despite these tremendous advances, tumor response to immunotherapy in unselected HCC patients was not commonly elicited, which led to the exploration of combination-based strategies to enhance efficacy. Vascular endothelial growth factor (VEGF) is related to inhibition of dendritic cells maturation *in vitro/vivo* through the activation of nuclear factor kappa-light-chain-enhancer of activated B cells (NF-κB), intratumoral accumulation of immunosuppressive cells of Tregs/MDSCs and inhibition of T-cell infiltration. Anti-VEGF treatment could help tumor vascular normalization and promote immune responses in the TME (75). In a phase Ib study of unresectable or advanced HCC, 31% HBV-infected patients treated with atezolizumab + bevacizumab achieved objective response, compared with 32% in the overall study population. After observing a tolerable safety profile and promising anti-tumor activity of this combination in phase 1b study, researchers carried out IMbrave150, a global, open-label, Phase 3, randomized study of atezolizumab + bevacizumab vs. sorafenib in unresectable HCC who had not received prior systemic therapy. The study demonstrated statistically significant and clinically meaningful improvement in overall survial and progression-free survival. In corresponding subgroup analysis, patients infected with HBV may profit more than non-virus HCC. Of note, atezolizumab + bevacizumab obviously prolonged median progression-free survival of HBV^+^ HCC comparing with sorafenib, but this phenomenon did not appear in the population of non-virus HCC (median progression-free survival, HBV^+^ HCC: 6.7 m vs. 2.8 m; non-virus HCC: 7.1 m vs. 5.6 m) (70). In another phase Ib study, focused on lenvatinib + pembrolizumab, a large proportion of patients with unresectable HCC experienced a long-lasting reduction in tumor size, including the HBV-infected patients (71). The combination of two powerful immunotherapy drugs was another trend of HCC treatment. Simultaneously the strong effect of blocking both PD-L1 and CTLA-4 pathway (durvalumab + tremelimumab) was also observed in unresectable HCC but not in HBV infected subgroup. Only 1 HBV infected patients (9%) achieved partial response status while 25% had an objective response in total population (66). The nivolumab + ipilimumab (anti-CTLA-4) combination therapy also presented clinically meaningful responses: Overall, ORR: 31% (7 had a CR); DCR: 49%; 24-month overall survival rate: 40% (68).

**Figure 2:**
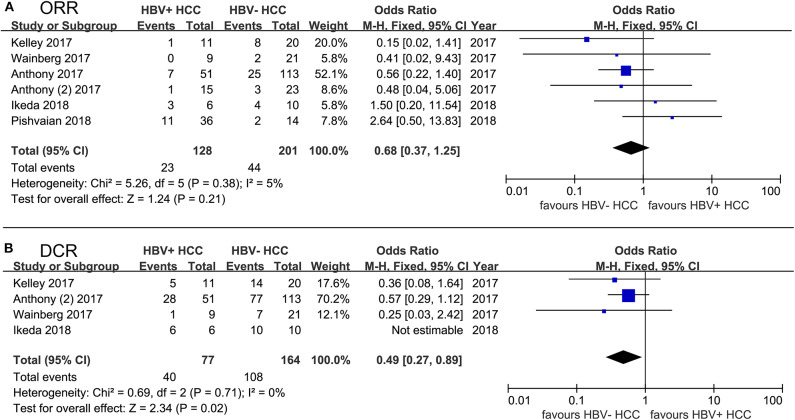
Forest plot of 6 studies evaluating the difference of PD-1/PD-L1 blockade efficacy between HBV^+^ HCC and HBV^−^ HCC. **(A)** using ORR to evaluate drug efficacy; **(B)** using DCR to evaluate drug efficacy. HCV^+^ HCC patients were excluded from the group of HBV^−^ HCC. Statistical analyses were performed using Review Manager (RevMan) software version 5.2 (Cochrane Collaboration). Pooled odds ratios (ORs) and 95% CIs were computed using the fixed-effects model.

We performed a pooled analysis to assess the different efficacy of PD-1/PD-L1 inhibitor therapy, including mono- or combined therapy, in both HBV^+^ HCC and HBV^−^ HCC patients. Of note, HCV^+^ HCC patients were excluded from the group of HBV^−^ HCC, and HBV^−^ HCC could be considered as non-viral HCC. We carried out a comprehensive systematic search for published literature in the PubMed and EMBASE databases. Additionally, we acquired partial data from posters, presentations, and meeting abstracts of the American Society of Clinical Oncology (ASCO) and the European Society of Medical Oncology (ESMO). The search strategy and selection criteria for our pooled analysis are provided in “Supplemental Methods.” We extracted or calculated odds ratios (ORs) to evaluate the strength of the association between drug efficacy and HBV infection. The *I*^2^ statistic was applied to evaluate the heterogeneity of studies in the pooled analysis and to help choose an appropriate model (*I*^2^ < 50%: fixed-effects model, *I*^2^ ≥ 50%: random-effects model). As expected, HBV^+^ patients achieved ORRs comparable to those of HBV^−^ HCC patients (OR, 0.68; 95% confidence interval [CI], 0.37–1.25; *P* = 0.21; Figure 2), and similar results were obtained in the monotherapy and combined-therapy subgroups (data not shown). However, the DCRs of HBV^+^ patients were significantly lower than those of HBV^−^ ones with PD-1/PD-L1 inhibitor therapy (OR, 0.49; 95% CI, 0.27–0.89; *P* = 0.02; Figure 2), and we observed stable disease more in HBV^−^ patients than in HBV^+^ patients, although not significantly (42% vs. 38%). In the subgroup of combined-therapy, the DCRs of HBV^+^ patients were also significantly lower than those of HBV^−^ ones (OR, 0.52; 95% CI, 0.27–0.99; *P* = 0.05). Additionally, we compared drug efficacy of HCV^+^ HCC with those in HBV^+^ HCC and HBV^−^ HCC. No significant difference was found in ORRs and DCRs of HCV^+^ HCC compared to those in HBV^+^ HCC and HBV^−^ HCC (all *P* > 0.05, Supplemental Table 1).

### Antiviral Effect of PD-1/PD-L1 Blockade

One study showed that adefovir monotherapy could control HBV viremia but HBsAg seroconversion was not achieved in patients with chronic hepatitis B, while the decreased but remaining number of peripheral Tregs still expressed high levels of PD-1 (76). In an animal model, viremia and antigenemia in woodchucks infected with woodchuck hepatitis virus (WHV) could be controlled via combination therapy containing anti-PD-L1 antibody and entecavir (77). Moreover, this combined strategy was better than antiviral treatment alone and incurred no liver damage AEs. PD-1/PD-L1 blockade led to significant HBsAg decline from baseline in virally suppressed chronic hepatitis B, and existed anti-HBV effect of PD-1/PD-L1 blockade was proved (78). There were some researches explored the antiviral effect of PD-1 blockade in virally infected HCC patients (57, 58). To achieve this goal, researchers assessed serum HBV surface antigen (HBsAg) and anti-HBs from HBV infected patients at baseline and after treatment. Existed but limited antiviral activity was found after treatment with nivolumab. No HBV reactivation was observed, and no patients infected with HBV achieved anti-HBs seroconversion, either (57). Similar results were found in HCV-infected patients, none of whom achieved a sustained virological response such as a reduction in HCV RNA levels lasting for 24 weeks (57). In these clinical studies, inclusion criteria included limitation of viral load (e.g., <100 IU/mL) by treatment of antiviral therapy. Thus, the anti-virus effects of checkpoint inhibitors are still in doubt and must be identified.

### Potential Signature for Predicting Drug Response

HCC heterogeneity is an important influence factor on immunotherapy effects; therefore, a potential predictive biomarker could help select patients who will probably benefit from the therapy.

PD-L1, a key check-point molecule, is generally expressed on tumor cells and interact with PD-1 to cause immune tolerance and evasion in TME (79). High PD-L1 expression in tumor cells, peritumoral hepatocytes and peripheral blood are associated with worse prognosis (80–82). And PD-L1 overexpression is positively related to higher levels of AFP, vascular invasion and poor tumor differentiation (81). Upregulating expression of *PD-1* and *PD-L1* in tumor-infiltrating effector CD8^+^ T cells is also relevant to disease progression and higher recurrence rates (44, 83). In a sense, the PD-L1 expression of tumor could be considered as an index to evaluate the immunosuppression caused by PD-1/PD-L1 axis and predict the efficacy of anti-PD-1/PD-L1 therapy. The predictive role of PD-L1 expression has been identified in the treatment of non-small-cell lung cancer (NSCLC) and gastric cancer with pembrolizumab (60). In HCC treated nivolumab, higher ORR was observed in HCC patients with PD-L1 expression on at least 1% of tumor cells, although not significantly (26% vs. 19%) (57). A similar outcome occurred in Keynote 224, response to anti-PD-1 therapy (pembrolizumab) was not associated with PD-L1 expression on tumor cells assessed retrospectively by immunohistochemistry. Of note, combined positive score (CPS) used for assessing not only tumor cells' PD-L1 expression but also those in immune cells, was significantly related to ORR and progression-free survival in this study (60). In Checkmate 459, the consistent effect was observed in patients with first-line nivolumab therapy compared to patients treated with sorafenib. And there was a trend toward better overall survial and ORR in patients with PD-L1 ≥ 1% (56). Thus, the PD-L1 expression related score model has the potential to be a reliable predictor of response to anti-PD-1 therapy.

Interestingly, another study showed that serum soluble PD-L1 (sPD-L1) concentration was several-fold higher in HBV-related HCC than in healthy control, a significant difference, while sPD-L1 was positively correlated with tumor PD-L1 expression (84). In addition, higher pre-treatment serum sPD-L1 levels were unfavorable predictors of worse disease-free and overall survival. Zeng et al. suggested that higher PD-L1 levels in peripheral blood was associated with a higher rate of tumor recurrence and progression (85). Because the existing clinical trials of anti-PD-1/PD-L1 immunotherapy did not provide enough data on PD-L1 expression in HBV-related HCC subgroup, the potential predictive role of PD-L1 should be further investigated in future studies. Based on previous promising observations, we hypothesize that PD-L1 is likely to serve as a predictor for drug efficacy or prognosis in HBV-related HCC.

## Related Mechanisms of Disrupting PD-1/PD-L1 Blockade Efficacy in HBV^+^ HCC

### HBV Induced Immune Cell Dysfunction and a Decline in Immune Cell Quantity

Using intracellular cytokine staining, researchers observed an inverse correlation between levels of T-cell response and viremia levels, implying that decreasing the viremia levels may have a role in enhancing immune responses of T cells (12). A recent study suggested that patients with low HBV levels have a signature of activated T-cell proliferation (27). Antiviral treatment could alter T-cell function, as CD8^+^ tumor-infiltrating lymphocytes from patients who received antiviral treatment (entecavir) did express higher effector T-cell markers and lower T-cell exhaustion markers. Antiviral drugs could activate T cells and strengthen their immune function, resulting in a better prognosis and decreased recurrence rates of HBV-related HCC (27, 86, 87). Peripheral expansion of Treg levels was also negatively correlated with HBV viral load, and the percentage of Tregs expressing PD-1 was significantly decreased when HBV replication was controlled (76). Thus, well-controlled HBV replication was of great importance to restoring immune system function.

### Altered Expression of Drug Targets: PD-1/PD-L1

PD-1 or PD-L1 blockade was applied to break the interaction of PD-1/PD-L1; therefore, sufficient enrichment of PD-L1 in tumor and host cells or PD-1 in immune-related cells was necessary for therapy to be efficacious. A single-nucleotide polymorphism (SNP) based experiment suggested that PD-1 mRNA levels in peripheral blood of HBV-infected patients were sequentially increased from *PD-1* rs10204525 genotypes GG and AG to AA and that the levels in genotype GG were significantly lower than in genotype AA; however, the results could not be found in the virus-negative group (88). Immunohistochemical (IHC) scores of PD-1 expression in tumor tissues and adjacent tissues from HCC patients with *PD-1*^+^ rs10204525 genotype AA were significantly higher than those from patients with genotypes AG and GG. The same results were observed in liver tissues of cirrhotic patients. PD-1 mRNA levels in peripheral-blood nuclear cells were found to be sequentially increased with the elevation of HBV DNA levels (89). The genotype determination of *PD-1* rs10204525 might become a potential biomarker of response to anti-PD-1/PD-L1 therapies.

### Tumor T-cell Receptor (TCR) Diversity

Virus-specific T cells were thought to be protective factors in the process of HBV infection, as patients with chronic HBV infection always had insufficient and dysfunctional HBV-specific T cells. Therefore, researchers engineered T cells to express specific HBV-specific TCRs through messenger RNA (mRNA) electroporation, enabling them to lyse HBV-producing hepatoma cells *in vitro*. *In vivo*, HBV-specific T cells expressing either the HBV-specific envelope or core TCR complex led to an 80–90% reduction of progressive viremia in mice after injections 3 ×/day for 12 days; longitudinal changes in viremia relative to baseline were determined at days 4, 8, and 12 (90). That is to say, T cells with specific TCRs were confirmed as targeting their corresponding antigens. In HBV-related HCC, data from high-throughput sequencing (HTS) technology showed that TCR diversity in tumors was higher than in adjacent non-tumor tissues. Limited overlap and rarely shared HCC clonal neo-antigens were found between these two kinds of tissues (24). Recently, autologous T cells were engineered to express TCR-specific epitopes from integrated HBV DNA in order to achieve antitumor efficacy in HBV-associated HCC. In addition, engineering HBV-specific T cells based on HBV transcriptomes of HCC cells was thought to be useful for personalized immunotherapy (91).

## Side Effect Evaluation of Anti–PD-1/PD-L1 Therapy

### Identifying Treatment-related Adverse Events (TRAEs)

When making treatment decisions for patients with advanced cancer, the oncologists should take the drug-related side effect and toxicity into consideration and adjust treatment strategy to decease the occurrence of TRAEs and mortality risk. Anti–PD-1/PD-L1 pathway therapy has been identified as safe in several advanced cancers including melanoma and NSCLC, and a systematic meta-analysis revealed that PD-1/PD-L1 inhibitor was in lower risk and better tolerated by comparing incidences of all and high-grade adverse events (AEs) between the PD-1/PD-L1 inhibitors group and the chemotherapy group (92). We assessed the safety of PD-1/PD-L1 inhibitor blockade based on data from about a dozen clinical trials (Table 2) to show that PD-1/PD-L1 blockade provided a similar and accepted safety profile for advanced-HCC patients vs. those with other malignant tumors. The most common TRAEs of PD-1/PD-L1 blockade monotherapy or combination therapy were fatigue, rash, pruritus, diarrhea, nausea, and increased levels of aspartate aminotransferase (AST) and alanine aminotransferase (ALT). Similar to previous toxicity assessments in other malignant tumors, TRAES were concentrically distributed across the categories of fatigued physical function, dermatological signs and elevated laboratory indices for liver function. Increased AST and ALT levels occurred more frequently than other Grade ≥3 TRAEs.

**Table 2:** Safety and tolerability of PD-1/PD-L1 monotherapy or combination therapy in total HCC patients and HBV^+^ subgroup.

**Drug**	**ClinicalTrials.gov number**	**Target**	**HBV^**+**^ HCC (%)**	**Safety evaluation in HBV^**+**^ HCC**	**All-grade TRAEs**	**Grade ≥3 TRAEs**	**All-grade TRAES (details)**	**Grade ≥3 TRAEs (details)**	**References**
Nivolumab	NCT01658878 (escalation phase)	PD-1	15 (31%)	Comparable symptomatic TRAEs to total HCC; No new safety signals	40 (83%)	12 (25%)	*Rash (23%), AST increase (21%), lipase increase (21%), pruritus (19%), amylase increase (19%), ALT increase (15%), diarrhea (10%), decreased appetite (10%)	Lipase increase (13%), AST increase (10%), ALT increase (6%), amylase increase (4%), fatigue (1%), anemia (1%)	(57)
Nivolumab	NCT01658878 (expansion phase)	PD-1	51 (24%)	Comparable symptomatic TRAEs to total HCC; No reactivation of HBV; No instances of anti-HBs seroconversion; No new safety signals	—	40 (19%)	Comparable to that observed in the dose-escalation phase	—	(57)
Nivolumab	NCT02576509	PD-1	116 (31%)	—	—	81 (22%)	*Skin (28%), hepatic (17%), endocrine (13%), Gastrointestinal (9%), Hypersensitivity/infusion reaction (8%)	*Hepatic (10%), Skin (2%), Gastrointestinal (2%)	(56)
Nivolumab	—	PD-1	—	—	—	2 (6%)	—	Bullous lichenoid drug eruption (3%), hepatitis (3%)	(59)
Nivolumab	—	PD-1	56 (74%)	No observed HBV reactivation	—	2 (3%)	Liver dysfunction (21%), pruritus (16%), anorexia (16%), nausea (13%), fatigue (8%)	liver dysfunction (1%), diabetes (1%)	(58)
Pembrolizumab	NCT02702414	PD-1	22 (21%)	No cases of flares of HBV; Few immune-mediated hepatitis	76 (73%)	27 (26%)	*Fatigue (21%), increased AST (13%), pruritus (12%), diarrhea (11%), rash (10%)	Increased AST (7%), fatigue (4%), increased ALT (4%)	(60)
Pembrolizumab	NCT02702401	PD-1	—	No cases of HBV flare	—	—	—	—	(61)
Camrelizumab (SHR-1210)	NCT02989922	PD-1	181 (83%)	High HBV infection rate (84%); Similar safety profile with total HCC	—	47 (22%)	*RCEP (67%), increased AST (25%), increased ALT (24%), proteinuria (23%), increased Blood bilirubin (17%)	*Increased AST (5%), decreased neutrophil count (3%)	(64)
BGB-A317	NCT02407990	PD-1	—	—	2 (18%)	0 (0%)	Fatigue (9%), rash (9%)	—	(63)
Cemiplimab	NCT02383212	PD-1	—	—	—	—	Fatigue (27%), decreased appetite (23%), increased AST (23%), abdominal pain (23%), pruritus (23%), dyspnea (23%)	—	(62)
Durvalumab	NCT01693562	PD-L1	9 (23%)	—	32 (80%)	8 (20%)	*Fatigue (28%), pruritus (25%), increased AST (23%), decreased appetite (13%), increased ALT (10%), diarrhea (10%), nausea (10%)	*Increased AST (8%), Increased ALT (5%)	(65)
Atezolizumab + bevacizumab	NCT02715531	PD-L1 + VEGF	51 (50%)	No new safety signals	84 (82%)	30 (27%)	Decreased appetite (28%), fatigue (20%), rash (20%), pyrexia (20%)	Hypertension (10%)	(69)
Atezolizumab + bevacizumab	NCT03434379	PD-L1 + VEGF	164 (49%)	—	276 (84%)	117 (36%)	*Hypertension (nearly 30%); diarrhoea, decreased appetite, pyrexia, increased ALT (all > 10%)	*Hypertension (10%)	(70)
Pembrolizumab + lenvatinib	NCT03006926	PD-1 + VEGF	8 (27%)	No unexpected safety signals	28 (93%)	18 (60%)	Decreased appetite (53%), hypertension (53%), diarrhea (43%), fatigue (40%), dysphonia (30%), proteinuria (30%)	*Hypertension (17%), increased AST (17%), WBC count decreased (13.3%), hyponatremia (10%)	(71)
Nivolumab + ipilimumab	NCT01658878	PD-1 + CTLA-4	—	—	—	148 (37%)	Pruritus, rash (data not shown)	—	(68)
Durvalumab + tremelimumab	NCT02519348	PD-L1 + CTLA-4	11 (28%)	TRAEs: Pruritus (27%), diarrhea (27%), increased ALT (27%), increased AST (27%), increased lipase (18%), rash (18%), increased amylase (18%), pancreatitis (9%) No unexpected safety signals	26 (65%)	10 (25%)	Fatigue (28%), pruritus (23%), increased ALT (20%), increased AST (18%), increased lipase (15%)	Increased AST (10%), increased lipase (10%), increased ALT (5%)	(66)
Durvalumab + tremelimumab	NCT02821754	PD-L1 + CTLA-4	—	—	—	—	—	—	(67)

Data are expressed as n (%) or event (%). TRAE, treatment-related adverse event; TRSAEs, treatment-related serious adverse event; AST, aspartate aminotransferase; ALT, alanine aminotransferase; RCEP, reactive cutaneous capillary endothelial proliferation; DT, discontinued treatment. *partial AEs with highest incidence are displayed.

### Safety Assessment in Conditions of HBV Infection

A study combining PD-L1 and CTLA-4 blockade showed that most TRAEs in HBV^+^ HCC were dermatological signs and elevated laboratory indices for liver function, including pruritus, rash, increased ALT level, and increased AST level (66). Of immune-related AEs induced by immune checkpoint blockade, dermatological toxicity, including rash and pruritus were the most common, representative and earliest-onset (93). Total prior data for HCC (both HBV^+^ and HBV^−^) indicated that dermatological toxicity was common but mild in anti–PD-1/PD-L1 therapy (Table 2), and the symptoms could be controlled by classical topical corticosteroid creams, peroral antipruritics or intravenous corticosteroid in more-severe cases.

Unlike the usual treatment-related symptoms, hepatic safety events should be emphasized in viral-infected subjects. Studies show that changes in AST and ALT levels are not consistent with typical radiographic liver findings; therefore, regular monitoring of liver function is important (94). AST and ALT, sensitive markers of acute hepatocyte damage, are frequently monitored in HBV-infected HCC with PD-1/PD-L1 blockade. And previous studies suggested that lack of prophylactic antiviral therapy was the most critical risk factor which contributed to HBV reactivation (95). To decrease the incidence of related AEs, HBV-infected patients involved in cohort studies were required to receive effective antiviral therapy to reach a low viral load, such as <100 IU/mL (57, 60). Fortunately, in contrast with prior CTLA-4 based research, HBV-infected patients who received nivolumab treatment did not demonstrate any new or unique safety signals (57). Because of controllable liver dysfunction in HCC patients and regular supervision of transaminase indicators, few patients suffered immune-mediated hepatitis and no HBV or HCV viral flares were observed in patients receiving anti-PD-1/PDL1 therapy (57, 60). Symptomatic TRAEs comparable to those reported in existing studies were found in patients with and without HCV or HBV infection. That is to say, HCC patients with well-controlled HBV viral risk factors could obtain safety profiles similar to those of non-infected participants and their risk of fulminant hepatitis could be lowered considerably.

## Conclusions

The HBV infection–related HCC immunosuppressed tumor microenvironment features upregulation of PD-1 in CD8^+^ T cells; long-lasting inhibition of Tregs via higher levels of IL-10 and TGF-β secretion; and the co-inhibitory signal of LAG3, TIM-3, and CTLA-4. Along with the above immunosuppressive factors, the PD-1/PD-L1 signaling pathway is thought to be the most important and widely adopted mechanism in the diagnosis and treatment of HCC patients with HBV infection.

In this study, we evaluated the safety and efficacy of PD-1/PD-L1 blockade immunotherapy, and also summarized the general differences between HBV related HCC and virus unrelated HCC (Table 3). Similar to patients with other tumor types, we observed acceptable toxicity and promising outcomes in HCC patients treated with PD-1/PD-L1 antibodies. According to our pooled analysis, ORRs of HBV^+^ HCC patients treated with anti-PD-1/PD-L1 immunotherapy were comparable to those of HBV^−^ ones, whereas DCRs of HBV^+^ patients were significantly lower. Based on our results, PD-1/PD-L1 blockade seemed to achieve slightly worse efficacy in HBV^+^ HCC patients than in HBV^−^ HCC ones. Nevertheless, when they were receiving a combination of anti–PD-1/PD-L1 and VEGF therapy, HBV-related HCC patients achieved ORRs and DCRs as high as those of HBV^−^ patients. However, even the existing but limited antivirus effect was found due to the HBV serological indicator. Due to well-controlled viral loads and routine antivirus therapy in current studies, the antiviral activity of anti–PD-1/PD-L1 therapy should be identified in a specially designed study in the future. The predictive value of PD-L1 expression for PD-1 blockade efficacy has been identified in not only melanoma and NSCLC but also HCC (57, 60, 99). PD-L1 expression level is commonly higher in HBV-related HCC, and the predictive effect of PD-L1 expression requires investigation in HBV-related cohorts.

**Table 3:** General differences between HBV related HCC and virus unrelated HCC.

**Aspect**	**HBV^**+**^ HCC's characteristics compared to virus unrelated ones**	**References**
**Disease state/feature**		
HBV itself	Strong variability; Viral heterogeneity accumulation; Hard to eradicate	(96)
HBV-induced mechanism	Chronic inflammation; Immune-mediated hepatocyte damage; higher rate of chromosomal alterations; p53 inactivation; WNT/b-catenin pathway inactivation; Oncogenic HBx protein; Insertional mutagenesis; Genomic instability	(97, 98)
Tumor microenvironment	Activated PD-1/PD-L1 signaling pathway; Co-inhibitory signal of LAG3, TIM-3, and CTLA-4; Exhausted CD8^+^ T cells; Immunosuppressive role of Tregs; Function-suppressive Trms. Higher levels of IL-10 and TGF-β secretion	(22, 26–36)
**Performance when treated with PD-1/PD-L1 immunotherapy**		
Efficacy	Comparable ORR and lower DCR (pooled analysis)	
Safety profile and toxicity	Comparable symptomatic TRAEs to total HCC; No reactivation of HBV; No cases of flares of HBV; No instances of anti-HBs seroconversion; Few immune-mediated hepatitis	(57, 60, 61, 64)
New safety signal	No new safety signals (consist with virus unrelated HCC)	(57, 66, 69, 71)
Antiviral effect	Existed anti-HBV effect of PD-1/PD-L1 blockade in previous study; No HBV reactivation in HBV^+^ HCC with low viral loads	(57, 58, 60, 78)

*LAG3, lymphocyte-activation gene 3; TIM-3, T-cell immunoglobulin and mucin domain-3; CTLA-4, cytotoxic T lymphocyte associated antigen-4; Tregs, regulatory T cells; Trms, resident memory T cells; ORR, objective response rate; DCR, disease control rate; TRAE, treatment-related adverse event*.

Factors, including HCC heterogeneity, HBV replication, and drug target, were related to disruption of PD-1/PD-L1 blockade efficacy in HBV^+^ HCC patients. Therefore, researchers should consider adjusting the cure strategy when the underlying mechanism is further clarified and explained. Finally, there remains a lack of valuable predictive biomarkers to monitor treatment effectiveness or choose the right patients to benefit from anti-PD-1/PD-L1 blockade immunotherapy, and further studies in these areas must be carried out in the future.

## Author Contributions

BL, WF, and WJ contributed to the conception, design, and final approval of the submitted version. BL, CY, XC, QF, HZ, and LL contributed to the collection and interpretation of data. ZT, YZ, PZ, and JZ contributed to the completion of figures and tables. BL, CY, and WF contributed to writing the paper. All the authors have read and approved the final manuscript.

## Conflict of Interest

The authors declare that the research was conducted in the absence of any commercial or financial relationships that could be construed as a potential conflict of interest.
