# Supplementary material for: Anti–PD-1/PD-L1 Blockade Immunotherapy Employed in Treating Hepatitis B Virus Infection–Related Advanced Hepatocellular Carcinoma: A Literature Review
Source: Front Immunol. 2020 May 28;11:1037. doi: 10.3389/fimmu.2020.01037 (PMC7270402; doi:10.3389/fimmu.2020.01037)
Supplement: Supplementary file 1 [file Table_1.docx]

**Supplemental Table 1: Characteristics of included studies for pooled analysis of PD-1/PD-L1 inhibitor therapy efficacy evaluation.**

|  |  |  |  | Objective response | | | Disease control | | |  |
| --- | --- | --- | --- | --- | --- | --- | --- | --- | --- | --- |
| Author (year) | Drug | ClinicalTrials. gov number | Target | HBV^+^ HCC | HCV^+^ HCC | HBV^−^ HCC | HBV^+^ HCC | HCV^+^ HCC | HBV^−^  HCC | Ref. |
| Anthony 2017 | Nivolumab | NCT01658878 (expansion phase) | PD-1 | 7 (14%) | 10 (20%) | 25 (22%) | 28 (55%) | 33 (66%) | 77 (68%) | (57) |
| Anthony 2017 | Nivolumab | NCT01658878 (escalation phase) | PD-1 | 1 (7%) | 3 (30%) | 3 (13%) | — | — | — | (57) |
| Wainberg 2017 | Durvalumab | NCT01693562 | PD-L1 | 0 (0%) | 2 (25%) | 2 (10%) | 1 (11%) | 5 (63%) | 7 (33%) | (65) |
| Kelley 2017 | Durvalumab + tremelimumab | NCT02519348 | PD-L1 + CTLA-4 | 1 (9%) | 1 (11%) | 8 (40%) | 5 (45%) | 4 (44%) | 14 (70%) | (66) |
| Pishvaian 2018 | Atezolizumab + bevacizumab | NCT02715531 | PD-L1 + VEGF | 11 (31%) | 10 (43%) | 2 (14%) | — | — | — | (69) |
| Ikeda 2018 | Pembrolizumab + lenvatinib | NCT03006926 | PD-1 + VEGF | 3 (50%) | 4 (40%) | 4 (40%) | 6 (100%) | 10 (100%) | 10 (100%) | (71) |

Data are n (%). HBV^−^ HCC: HBV^−^ HCV^−^ HCC (non-viral HCC); PD-1, programmed cell death protein 1; PD-L1, programmed death-ligand 1. CTLA-4: cytotoxic T lymphocyte associated antigen-4; VEGF: vascular endothelial growth factor.
