# Supplementary material for: Anti–PD-1/PD-L1 Blockade Immunotherapy Employed in Treating Hepatitis B Virus Infection–Related Advanced Hepatocellular Carcinoma: A Literature Review
Source: Front Immunol. 2020 May 28;11:1037. doi: 10.3389/fimmu.2020.01037 (PMC7270402; doi:10.3389/fimmu.2020.01037)
Supplement: Supplementary file 2 [file Data_Sheet_1.docx]

***Pool-analysis***

**Search strategy**

Eligible literatures updated until September of 2019 were collected using “(PD-1 or programmed cell death-1 or nivolumab or pembrolizumab or PD-L1 or programmed death ligand-1 or atezolizumab) AND (HCC or hepatocellular carcinoma or liver cancer) AND Clinical trial as the keywords. The reference list was also checked for relevant articles.

**Selection criteria**

**Inclusion criteria:**

- Randomized controlled trials;
- Hepatocellular carcinoma was diagnosed by pathological examination;
- The study should contain subgroup of HBV infected patients;
- The rates of objective response or disease control of HBV+ HCC could be obtained from the literature;
- If there were duplicate articles, only the most complete or latest one could be included.

**Exclusion Criteria:**

- Studies without sufficient data for estimating odds ratio (OR) and 95% confidence interval (CI);
- Nonhuman research
- Other tumor types
